# In Vitro and In Vivo Characterization of the Transdermal Gel Formulation of Desloratadine for Prevention of Obesity and Metabolic Syndrome

**DOI:** 10.3390/ph16040578

**Published:** 2023-04-12

**Authors:** Eman M. Mohamed, Sathish Dharani, Tahir Khuroo, Rania Hamed, Mansoor A. Khan, Ziyaur Rahman

**Affiliations:** 1 Irma Lerma Rangel College of Pharmacy, Texas A&M Health Science Center, Texas A&M University, College Station, TX 77843, USAmkhan@tamu.edu (M.A.K.); 2 Department of Pharmacy, Faculty of Pharmacy, Al-Zaytoonah University of Jordan, Amman 11733, Jordan

**Keywords:** desloratadine, metabolic syndrome, hydroxypropyl methylcellulose, transdermal gel, permeation, pharmacokinetic

## Abstract

Chronic use of antihistamines can induce abnormalities in lipid absorption with potential excessive accumulation of lipids in the mesentery that can lead to the development of obesity and a metabolic syndrome. The focus of the present work was to develop a transdermal gel formulation of desloratadine (DES) to prevent/reduce obesity and metabolic syndromes. Nine formulations were prepared to contain hydroxypropyl methylcellulose (2–3%), DES (2.5–5.0%), and Transcutol^®^ (15–20%). The formulations were evaluated for cohesive and adhesive properties, viscosity, drug diffusion through synthetic and pig ear skin, and pharmacokinetics in New Zealand white rabbits. Drug permeation was faster through the skin compared to synthetic membranes. The drug had good permeation, as indicated by very short lag time (0.08–0.47 h) and high flux (59.3–230.7 μg/cm^2^.h). The maximum plasma concentration (C_max_) and area under the curve (AUC) of transdermal gel formulations were 2.4 and 3.2 fold that of the Clarinex tablet formulation. In conclusion, as indicated by the higher bioavailability, transdermal gel formulation of DES may decrease the dose of the drug, compared to commercial formulation. It has the potential to reduce or eliminate metabolic syndromes associated with oral antihistamine therapy.

## 1. Introduction

Approximately 50% of Americans experience various types of allergies each year. It is the sixth leading cause of chronic illness in the USA. Examples of allergic conditions are asthma, anaphylaxis, hives, rhinitis, eczema, etc. [1,2,3]. Each year, allergies cost $18 billion in healthcare and food allergies cost $25 billion [4,5]. Pharmacological therapeutics are used for symptomatic management of allergic conditions, e.g., antihistamines, glucocorticoids, immunotherapy, epinephrine, etc. [6,7]. Among all pharmacologic therapeutics, antihistamines are most commonly used for seasonal allergies, rhinitis, urticaria, etc., due to their low cost, easy access as an OTC, incidence of adverse events, etc. Examples of antihistamines available as OTC are diphenhydramine, chlorpheniramine, cetirizine, loratadine, fexofenadine, and desloratadine. Antihistamines alleviate allergic symptoms by blocking histamine mediated effects primarily through the H_1_-receptor [8,9,10]. The availability of antihistamines as OTC lead to their misuse and abuse, especially those having a sedating effect, e.g., diphenhydramine [11]. Second and third generation antihistamines are less sedative, e.g., cetirizine and desloratadine (DES) [12,13]. Although antihistamines are generally considered safe, the published literature with data analysis raise concerns regarding their long-term effect on metabolic disorders, especially obesity [14].

The National Health and Nutrition Examination Survey investigated the association between prescription H_1_ antihistamine and obesity using data from 2005–2006. Body measurements, plasma glucose and insulin concentrations, and lipid levels were compared between subjects taking prescription antihistamines and control (age and gender matched). Significantly higher weight, waist circumference, and insulin concentration were found in subjects taking prescription H_1_ antihistamine than in matched controls. It was concluded that H_1_ antihistamine may contribute to the increased prevalence of obesity and metabolic syndrome [14]. Recently, the association between antihistamine use in children and obesity was reported. In a single-center cohort study on children, body mass index (BMI) was assessed. Of the 32 children in the study, 13 were antihistamine users while 19 were antihistamine naive. Antihistamine users have an increased BMI of 1.17 per year compared to a decrease by 0.06 per year in the control group. Furthermore, no significant difference in triglycerides, glucose, or liver enzymes was found [15]. Our lab investigated the molecular and cellular mechanism of antihistamine-induced metabolic syndrome. Chylomicrons, containing dietary lipids absorbed from the intestine, reach the blood through the mesenteric lymphatic network where mesenteric lymphatic vessels (MLVs) play a key functional role. Spontaneous phasic contractions of MLVs create forces that drive the lipid-enriched lymph through the mesenteric lymphatic network. The tone of MLVs plays an important role in the resistance of lymphatic vessels to lymph flow. Postprandial increases in gastrointestinal lymph formation, and flow inhibit phasic contractions and reduce tone in the MLVs in a shear-dependent manner. Histamine is responsible for the more prolonged components of flow-dependent post-prandial adaptation of MLVs contractility and tone [16,17,18]. Any dysfunction of the lymph flow-dependent regulatory mechanisms of lymphatic tone and contractility would alter the flow of lymph and, as a consequence, would worsen the postprandial absorption of lipids from the gut via lymph. The prolonged intake of antihistamines may alter the histamine-mediated components of flow-dependent post-prandial adaptation of MLV contractility and tone. Such alterations would likely lead to a chronic increase in MLV tone and, subsequently, to chronically increased MLV resistance to lymph flow. Therefore, chronic use of antihistamines could induce abnormalities in lipid absorption with the potential excessive accumulation of lipids in the mesentery as a starting point for the development of obesity and, subsequently, the development of a metabolic syndrome. We confirmed this hypothesis in rat studies. The DES-fed group showed a statistically significant increase (*p* < 0.05) in weight, fat accumulation mesenteric tissues, elevated tonic, and phasic contractility of MLVs as well as altered reactivity of mesenteric lymphatic vessels to postprandial increase in lymphatic flow, diminished endothelium-derived relaxing factors, high serum triglycerides with their routing towards portal blood, high serum HDL, high fasting blood glucose levels, and fat accumulation in the liver [19,20]. Antihistamine-associated metabolic syndrome can be eliminated/reduced by changing the route of administration. DES belongs to the BCS class I drugs category, and is slightly soluble in water and sparingly soluble in organic solvents [21]. In vitro studies of DES transdermal patches, topical ointments, and gel formulations were reported in the literature [22,23,24]. However, none of the reported works demonstrated skin permeation of the drug and or in vivo pharmacokinetics to demonstrate bioequivalency with the oral formulation of DES. Furthermore, the reported formulations are for the localized effect of the drug [22,23,24]. The objective of the study was to develop a transdermal gel formulation of DES for systemic administration in order to eliminate metabolic syndrome associated with its long-term use, e.g., obesity and diabetes. Transdermal gel formulation was developed and evaluated for physicochemical, permeation, and pharmacokinetic attributes.

## 2. Results and Discussion

Troyanova-Wood et al. reported detrimental metabolic effects of long-term oral intake of DES. Investigators recommended changing the route of administration of antihistamines from oral to topical to eliminate adverse metabolic effects [20]. Topical formulations of DES were reported in patent applications and the literature. Reported topical formulation consisted of 1–10% DES and 2–20% solubilizer such as hydroxypropyl β-cyclodextrin (HPBCD) and dimethyl-beta-cyclodextrin. Several studies have investigated the feasibility of developing intranasal/transdermal formulation of DES. Yurtdaş-Kırımlıoğlu et al. reported thermosensitive in-situ hydrogel for intranasal administration using a combination of Kolliphor^®^ 188 (K188) and Pluronic^®^ F127 (P127) as solubility improving agents. An in vitro release study from the in-situ hydrogel formulations was conducted using a cellulose acetate dialysis bag (MW cut off 12,000–14,000 Da). The hydrogel showed higher initial release owing to the burst effect, due to Kolliphor^®^ 188 compared to the drug alone [25]. Trividi et al. also reported a transdermal patch of DES developed using Hydroxypropyl methylcellulose (HPMC) [24]. An in vitro diffusion study was conducted using a cellophane membrane and patches exhibited controlled release permeation with zero order kinetics. None of the reported intranasal/transdermal formulations were evaluated for permeations and pharmacokinetics.

### 2.1. Preliminary Data

**Figure 1:**
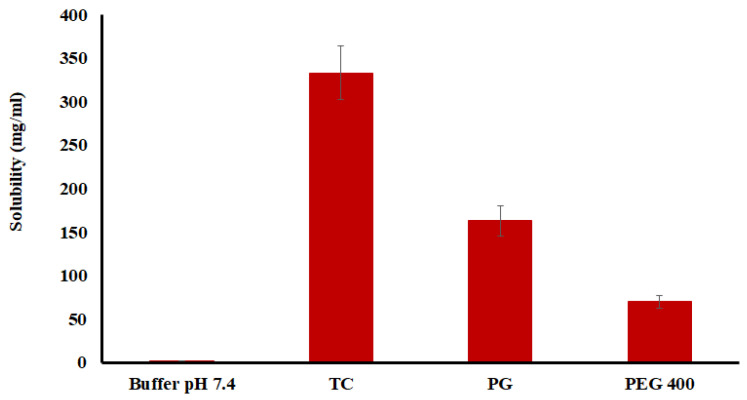
Solubility of desloratadine in various solvents. Data are shown as mean ± SD, *n* = 6.

As shown in Figure 1, the solubility of DES in phosphate buffer 7.4, Transcutol^®^, polyethylene glycol 400 (PEG 400), and propylene glycol (PG) was 1.5, 333.3, 70, and 163.3 mg/mL, respectively (Figure 1). Based on the solubility data, Transcutol^®^ was selected as a permeation enhancer for the gel formulation [26].

**Figure 2:**
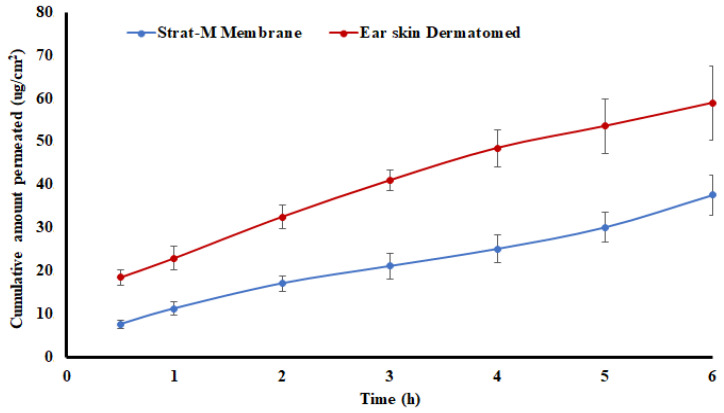
Permeation of saturated solution of desloratadine (in phosphate buffer pH 7.4) through Strat-M^®^ and pig ear skin. Data are shown as mean ± SD, *n* = 6.

The drug has good permeation, as indicated by immediate diffusion across the synthetic membrane. Furthermore, the permeation was faster through the pig skin compared to the synthetic membrane. The flux (J) value was 4.99 and 6.54 μg/cm^2^.h, respectively (Table 1 and Figure 2). A probable reason for the higher permeation of the drug through the pig skin may be due to biocomponents of the skin that aid in the drug transport across the membrane compared to the synthetic membrane. These components could be enzymes, lipids, and/or proteins, etc. [27,28]. The permeability under a steady state is referred to as the apparent permeation (P_app_) and can be calculated mathematically using flux and the initial drug concentration in the donor compartment [29]. P_app_ was 3.3 × 10^−3^ and 4.4 × 10^−3^ cm/h for synthetic membrane and pig skin, respectively.

**Figure 3:**
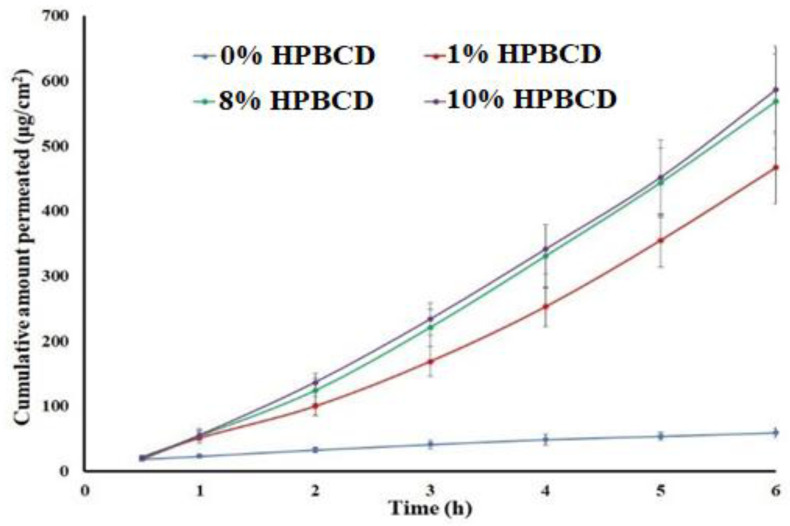
Effect of hydroxypropyl β-cyclodextrin on permeation of saturated solution of desloratadine (in phosphate buffer pH 7.4) through pig ear skin. Data are shown as mean ± SD, *n* = 6.

To maintain the sink condition in the receptor compartment, various percentages of HPBCD in the receptor medium were investigated. Increasing the HPBCD percentage in the receptor chamber resulted in a statistically significant (*p* < 0.05) increase in the permeated drug (Figure 3). The value of the J was 6.54, 91.98, 111.28, and 111.85 μg/cm^2^.h at 0, 1, 8, and 10% HPBCD, respectively. The increase in flux was 14.1, 17.0, and 17.1 folds at 1, 8, and 10% HPBCD, respectively, compared to without HPBCD in the receptor medium. Similarly, P_app_ values were 4.4 × 10^−3^, 61.3 × 10^−3^, 74.2 × 10^−3^, and 74.6 × 10^−3^ cm/h at 0, 1, 8, and 10% HPBCD, respectively. The increase in flux can be explained by the complexation of the drug and thus maintaining the drug in the solubilized form and sink condition [30,31]. The literature reported an increase in drug permeation through the skin in the presence of cyclodextrins. Cyclodextrins carry the drug through the aqueous barrier, from the bulk solution towards the lipophilic surface of biological membrane, where the drug molecules partition from the complex into the lipophilic membrane [30,31]. Thus, cyclodextrins increase drug availability at the barrier surface. A similar observation was reported for DES permeation [23].

### 2.2. Gel Characterization

**Figure 4:**
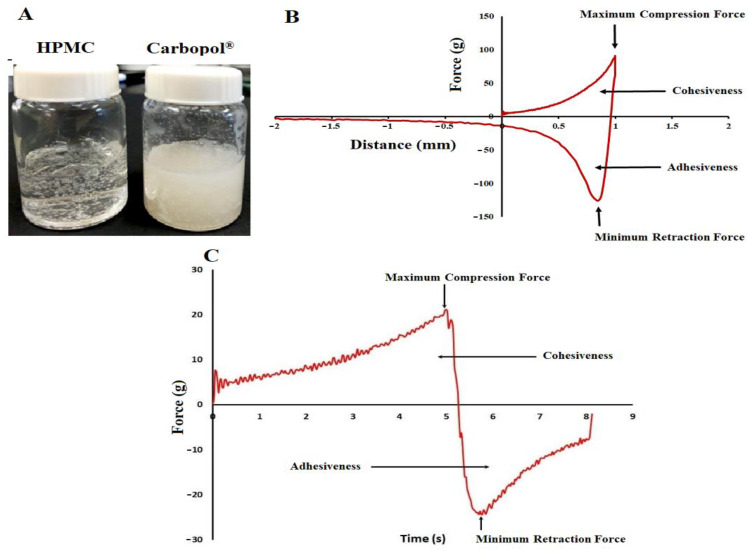
(**A**) Formulation of desloratadine hydrogel using various polymers. (**B**) Force distance profile of the gel. (**C**) Force time profile of the gel. Data are shown as mean ± SD, *n* = 6.

Gel was prepared using HPMC and Carbopol^®^ polymers. Carbopol^®^ requires the addition of an alkaline agent to cause ionization and unfolding of the polymer chain [32,33]. However, the addition of sodium hydroxide to the gel causes precipitation/crystallization of the drug since the drug has pH-dependent solubility [21], as shown in Figure 4A. HPMC polymer was selected for gel development since the pH adjustment is not required for gel preparation. HPBCD was also added to the gel formulation as DES is prone to oxidative degradation that resulted in the formation of an orange-yellow color degradant, N-formyl desloratadine [34]. Cyclodextrin is reported to reduce/inhibit this degradation if used in 1:5 to 1:25 ratios with respect to the drug to cyclodextrin. levels [35]. DES: HPBCD 1:5 was used in the gel formation to reduce/prevent N-formyl desloratadine formation.

The pH of the transdermal gel formations varies form 9.13–9.80. The pH of the gel without the drug varied from 7.40–8.19. The addition of the drug resulted in an increase in the pH of the formulation due to the drug’s alkaline nature [21]. The viscosity of the formulations varied from 31.9 ± 2.7 (F1) to 519.9 ± 48.3 (F6) Pas. The viscosity was affected by HPMC and Transcutol^®^ percentage in the formulation. As expected, HPMC increased while Transcutol^®^ decreased the viscosity of the formulations (*p* < 0.05).

### 2.3. Cohesiveness and Adhesiveness

The hardness, cohesiveness, and adhesiveness of the transdermal gel formulations were determined from the force-distance/time plot (Figure 4B,C). Hardness was deduced from the maximum compression force while cohesiveness measured the work required to deform the gel during downward movement. It was determined from the work needed to apply the gel. The area of the second curve represents the adhesiveness of the gels and is measured by the work conducted to remove the gel (Figure 4B,C) [36]. The hardness, cohesiveness, and adhesiveness of the gel formulations increased with an increase in polymer and drug percentage while these properties decreased with an increase in the Transcutol^®^ percentage. This was related to the viscosity of the formulation. The hardness of the gel formulations varied from 14.26 ± 1.7 (F1) to 21.66 ± 4.4 (F6) g. The cohesiveness and adhesiveness of the gel formulations varied from 8.8 ± 0.7 (F1) to 19.7 ± 2.1 (F6) g.mm and from 1.1 ± 0.9 (F9) to 12.6 ± 2.1 (F1) g.mm, respectively. F1 represented low polymer (2%) and drug (2.5%) levels and high Transcutol^®^ (20%) loading while F6 contained high polymer (3%) and drug (5%) levels and high Transcutol^®^ (15%) loading. Similarly, viscosity of the formulations varied from 31.9 ± 2.7 (F1) to 519.9 ± 48.3 (F6) Pas. Thus, the gel formulations had low values of adhesiveness and cohesiveness that make it easy to apply and remove them.

### 2.4. Lag Time

**Figure 5:**
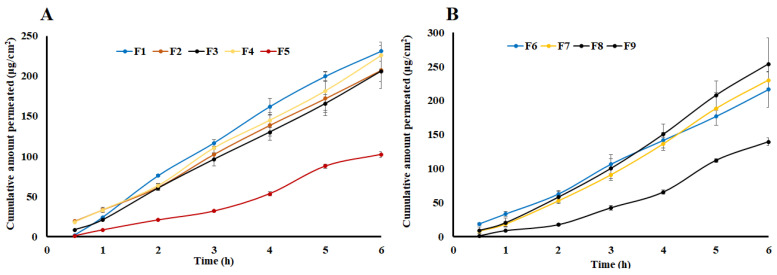
(**A**) Permeation of transdermal gel formulations F1, F2, F3, F4, and F5 through Strat-M^®^. (**B**) Permeation of transdermal gel formulations F6, F7, F8, and F9 through Strat-M^®^. Data are shown as mean ± SD, *n* = 6.

**Figure 6:**
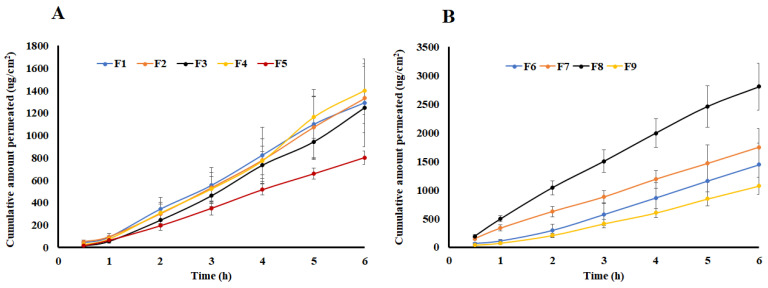
(**A**) Permeation of transdermal gel formulations F1, F2, F3, F4, and F5 through pig ear skin. (**B**) Permeation of transdermal gel formulations F6, F7, F8, and F9 through pig ear skin. Data are shown as mean ± SD, *n* = 6.

The lag time was calculated from 0.5–2 h region of the time-permeated drug curve (Figure 5 and Figure 6) where the curve was linear and did not reach a steady-state. The lag time of drug permeation was very short for both synthetic and skin membranes. It varied from 0.08 (F8) to 0.47 (F4) h for pig ear skin. Compared to the skin membrane, the lag time was longer for drug permeation through the synthetic membrane. It varied from 0.18 (F2) to 0.64 (F1) h (*p* > 0.05).

Moreover, the polymer increased the lag time while the drug and Transcutol^®^ percentage shorten the time for the drug to permeate through the skin. An increase in lag time with an increase in the polymer percentage in the gel formulation was due to an increase in the viscosity. Viscosity and diffusion have an indirect relationship. Increasing viscosity of the gel formulations resulted in an increase in the path length and tortuosity that the drug has to overcome to reach the absorptive site [37,38]. For instance, the lag time was 0.47 and 0.08 h for F4 and F8, respectively. F4 and F8 contained 3 and 2% of the polymer while the drug (5%) and Transcutol^®^ (20%) percentages were kept constant. Lag time was indirectly proportional to the drug and Transcutol^®^ percentage in the formulations. For example, the lag time of the gel formulations with 2.5 (F1) and 5% (F8) drug percentages were 0.40 and 0.08 h while Transcutol^®^ (20%) and the polymer (2%) percentages were kept constant. A decrease in the lag time with an increase in drug percentage in the gel formulations was due to an increase in the concentration gradient [39]. Similar to the drug percentage in the gel formulations, increasing Transcutol^®^ resulted in a decrease in lag time due its effect on the barrier properties of the skin. Transcutol^®^ acts as a drug permeation enhancer by diffusing into the stratum corneum and altering the solubility parameter of the intercellular lipid domain of the skin [40]. For example, F1 and F9 contained 20 and 15% Transcutol^®^ and exhibited 0.40 and 0.30 lag times, respectively. A similar trend was observed on the effect of the drug, polymer, and Transcutol^®^ percentage on the drug permeation through the synthetic membrane.

### 2.5. Flux and Apparent Permeation

The flux was calculated from the slope of the 3–6 h region of the permeated drug-time curve (Figure 5 and Figure 6). At this region of the plot, the curve was linear. As shown in Table 1, the J varied from 12.6 (F1) to 51.7 (F8) μg/cm^2^.h, and from 149.7 (F5) to 347.0 (F8) μg/cm^2^.h for Strat-M^®^ and pig ear skin, respectively (Table 1). A higher flux in the case of Strat-M^®^ membrane in comparison to pig ear skin (*p* < 0.05) can be explained by Transcutol^®^. Transcutol^®^ is known for altering skin permeability so that more drugs can diffuse through the skin [40]. The drug flux through the skin was 4.5–19.8 folds that of Strat-M^®^. Transcutol^®^ did not have a significant impact on the drug flux through the synthetic membrane.

Similar to lag time, polymer percentage decreased J while the drug and Transcutol^®^ percentage increased the drug flux through the membrane. The J values through pig ear skin were 302.5 and 347.0 μg/cm^2^.h for the F4 and F8 formulations, respectively. These two formulations contained 3 and 2% HPMC, respectively, while drug (5%) and Transcutol^®^ (20%) percentages were kept constant. Similarly, formulations F1 and F8 showed the effect of drug percentage on the flux. The flux values were 248.6 and 347.0 μg/cm^2^.h for F1 (2.5%) and F8 (5%), respectively. These formulations contained an identical amount of the polymer (2%) and Transcutol^®^ (20%). The effect of Transcutol^®^ was exhibited by the gel formulations F7 and F8. The formulations F7 and F8 contained 15 and 20% Transcutol^®^ in their composition, respectively, and corresponding flux values were 287.9 and 347.1 μg/cm^2^.h, respectively.

Apparent permeability followed trend of the flux. P_app_ values range from 0.6 × 10^−3^ to 1.7 × 10^−3^ and from 7.2 × 10^−3^ to 13.5 × 10^−3^ cm/h for synthetic and pig ear skin (*p* < 0.05), respectively (Table 1).

Data of in vitro permeation through the synthetic membrane were fitted data into zero-order, first-order, Higuchi, and Korsmeyer-Peppas models. The kinetic drug permeation through the synthetic membrane can be best described by the zero-order release model followed by the korsmeyer-Peppas and Higuchi diffusion models based on the determination coefficient value (R^2^) value [24,36].

### 2.6. Drug Retained in the Skin

**Figure 7:**
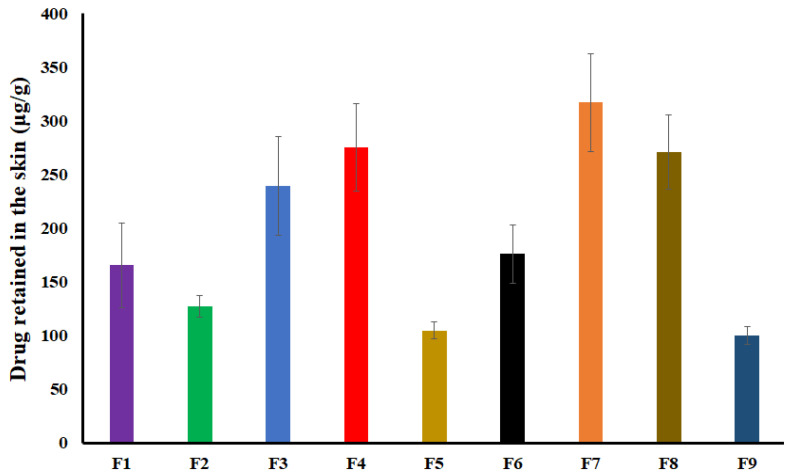
Drug retained in pig ear skin after the permeation experiment. Data are shown as mean ± SD, *n* = 6.

The drug retained (DR) in the skin after the permeation study varied from 0.35 ± 0.03% (104.8 ± 8.0 μg/g, F5) to 1.59 ± 0.23% (317.4 ± 45.5 μg/g, F7) (Figure 7).

The drug percentage in the formulations increased while the polymer and Transcutol^®^ percentage decreased the DR. The drug has to traverse through various barriers/steps from gel formulation before it reaches the systemic circulation, i.e., stratum corneum, dermis, and subcutaneous tissue (blood vessels). The polymer and Transcutol^®^ decrease DR by a different mechanism. HPMC decreased the drug diffusion from the formulation while Transcutol^®^ increased drug permeation from various layers of the skin. Increasing the drug percentage in the gels increased both drug diffusion and permeation by increasing the concentration gradient. For instance, F5, F3, and F8 have a DR of 0.35 ± 0.03 (104.8 ± 8.0 μg), 0.96 ± 0.19 (239.6 ± 46.3 μg), and 1.36 ± 0.17% (271.2 ± 34.5 μg), respectively. These formulations have 15, 2.5, and 3%; 2.5, 3.75, and 17.5’ and 20, 5.0, and 2% of Transcutol^®^, DES, and Polymer, respectively.

### 2.7. Pharmacokinetic Studies

**Figure 8:**
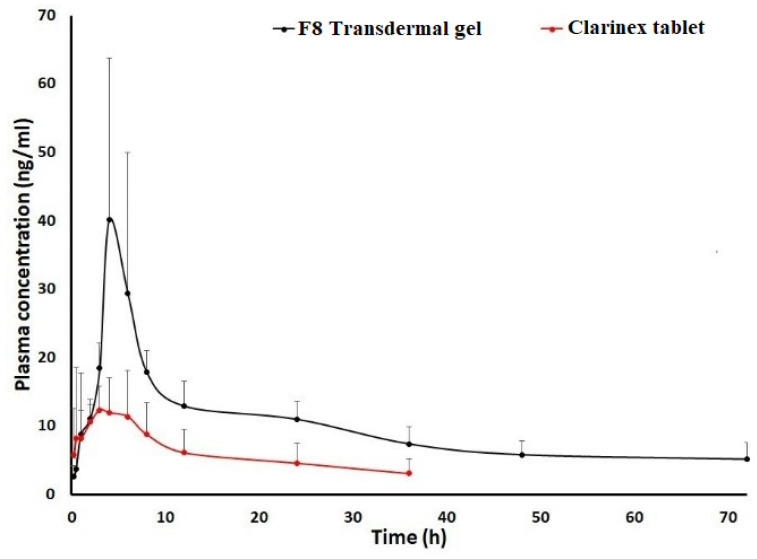
Pharmacokinetic profiles of F8 transdermal gel and Clarinex tablet formulations in New Zealand white rabbits. Data are shown as mean ± SD, *n* = 4.

DES belongs to the BCS class one drug category which means the drug is highly soluble and permeable [21,41]. The oral bioavailability of DES in humans is not reported in the literature. However, the oral bioavailability in male and female rats are reported as 50% and 95%, respectively [42]. The drug is rapidly absorbed after oral administration and food has no effect on the rate and extent of the absorption [43]. Pharmacokinetic profiles of oral and transdermal gel formulations were not superimposable (Figure 8). Furthermore, pharmacokinetic parameters were completely different in these formulations. The T_max_ (time to achieve C_max_) was 3.4 ± 2.3 and 4.3 ± 1.3 h for the tablet and the gel formulations, respectively. The reported T_max_ for DES is ≥3 h in humans [44]. The shorter T_max_ in the case of tablet formulation was due to the fact that the drug immediately enters into systemic circulation after absorption from the gastrointestinal tract. For the gel formulation, it has to cross various layers of the skin before entering into the systemic circulation. However, no statistical difference (*p* > 0.05) was observed between two formulations for T_max_. C_max_ (maximum plasma concentration) and AUC (area under the plasma concentration-time curve) were higher in the case of transdermal gel compared to tablet formulation. C_max_ and AUC were 45.2 ± 24.6 ng/mL and 1028.3 ± 385.7 ng/mL.h, and 19.0 ± 3.5 ng/mL and 318.7 ± 176.3 ng/mL.h for the gel and the tablet formulations, respectively. The gel formulation exhibited an increase of 2.4 and 3.2 fold of C_max_ and AUC, respectively, compared to the tablet formulation. Moreover, only AUC exhibited statistically significant difference between two formulations (*p* < 0.05). Higher values of C_max_ and AUC in the case of the transdermal formulation were due to the avoidance of first-pass metabolism. Orally administered DES is metabolized by the liver via cytochrome P450 [45]. The drug was detected during the period of the study (72 h) from both formulations. However, the drug concentration fell below the quantification limit (2.5 ng/mL) after 36 h in the case of the tablet formulation. Moreover, the plasma concentration stayed above >5 ng during the period of the pharmacokinetic study in the case of the transdermal gel formulation. Both formulations also exhibited other pharmacokinetic differences. The mean residence time (MRT) was longer and elimination was slow in the case of the transdermal formulation compared to the oral tablet. MRT measures the average time a drug stays in the body. The tablet and transdermal formulation of MRT were 26.3 ± 12.1 and 58.5 ± 17.8 h, respectively (*p* < 0.05). The elimination rate constant is the rate at which the drug is cleared from the body. Thus, it provides information on the elimination process in terms of whether it is slow or fast. The drug elimination was faster in the case of oral tablet formulation compared to the transdermal gel formulation as indicated by the terminal elimination rate constant value (λ_z_). λ_z_ values were 0.042 ± 0.012 and 0.019 ± 0.007 h^−1^ for the tablet and transdermal gel, respectively (*p* < 0.05). This indicated that the transdermal formulation avoided first metabolism, slow downed drug elimination from the body, and increased the bioavailability [46].

## 3. Materials and Methods

### 3.1. Materials

DES was purchased from Lide Pharmaceuticals Limited, Nanjing, China. Polyethersulfone membrane (Strat-M^TM^, Merck Millipore, Darmstadt, Germany). HPMC (Benecel^TM^ K100M PHARM), Carbopol^®^ 934P NF, Carbopol^®^ 980, Transcutol^®^ HP, and HPBCD (Kleptose, oral grade) were obtained from Ashland Specialty Ingredients (Wilmington, DE, USA); Lubrizol Advanced Materials, Inc., (Cleveland, OH, USA); Gattefosse SAS (Nanterre, France); and Roquette America Inc. (Keokuk, IA, USA), respectively. Deuterated DES (DES-d4) was obtained from Toronto Research Chemicals, Ontario, Canada. Heparinized rabbit plasma was purchased from BioChemed Services, Winchester, VA, USA. PEG 400, PG, potassium dihydrogen phosphate, orthophosphoric acid, sodium hydroxide, formic acid, and methanol were purchased from Fisher Scientific, Asheville, NC, USA. All reagents were of analytical grade and were used as received. In-house water (18 MU.cm, Millipore Milli-Q Gradient A-10 water purification system) was used in this work.

### 3.2. Methods

#### 3.2.1. Drug Solubility

The solubility studies were carried out by adding an excess quantity of the drug in a scintillation vial containing 5 mL of phosphate buffered pH 7.4 and sonicated for 5 min to achieve supersaturation. The samples were kept for shaking over a horizontal water path at 37 ± 0.5 °C for 24 h. The samples were centrifuged (Eppendorf 5430R, Eppendorf North America, Inc, Enfield, CT, USA) for 15 min at a speed of 5000 rpm followed by filtration through a 0.45 µm Millipore membrane. The filtrate was analyzed for DES by the validated HPLC method. The solubility experiment was carried out in triplicate.

#### 3.2.2. High Performance Liquid Chromatography Analysis

The HPLC equipment consisted of Agilent 1260 series (Agilent Technologies, Wilmington, DE, USA) equipped with a quaternary pump, online degasser, column heater, autosampler, and UV/Vis detector. Data collection and analysis were performed using Openlab software (Agilent Technologies, Wilmington, DE, USA). Separation of the drug was achieved on a 4.6 × 150 mm, 5 µm SunFire^TM^ C18 (Waters, SunFire Columns, Ireland) column and a C18, 4.6 × 2.5 mm (5 µm packing) Luna C18 guard column (Phenomenex, Torrance, CA, USA). The mobile phase consisted of a mixture of methanol and 20 mM phosphate buffer pH 7.0 (80:20, *v*/*v*) flowing at 1.0 mL/min. The column and auto-sampler were maintained at 30 °C. A sample volume of 10 μL was injected into the system and DES was detected at 210 nm. Two injections per sample were analyzed by HPLC to demonstrate the reproducibility of the data.

#### 3.2.3. Pig Skin and Synthetic Membranes Preparation

Porcine ears of young pigs were obtained from the HEB store. They were cleaned up in tap water followed by hair removal with a clipper. Strips of skin membranes (approximately 6 cm diameter) were cut at a thickness of 400–600 μm using an electric dermatome (Integra/Padgett Model B, Integra LifeSciences, Princeton, NJ, USA). Each membrane was wrapped in filter paper, moistened with saline solution, packed in aluminum foil, and frozen at −20 °C until use. The dermatome skin membranes can be used within 6 months of preparation [47]. The skin was thawed, washed with distilled water, and measured for integrity using a vapometer (Delfin Technologies, Ltd., Kuopio, Finland) that measures transepidermal water loss (TEWL) in a closed chamber system. All the TEWL values were recorded in ambient conditions (25 ± 5 °C, 50 ± 5% RH). The skin samples that showed TEWL of ≤15 g/m^2^/h were used in the permeation study [48,49].

#### 3.2.4. Transdermal Gel Preparation

DES and HPBCD were dissolved in Transcutol^®^ in a ratio of 1:5 by sonication. Separately, HPMC was dissolved in water by stirring overnight. The drug solution and polymer gel were mixed with stirring, packed in an aluminum tube, and stored at ambient conditions. Nine formulations were prepared that contained HPMC (2–3%), DES (2.5–5.0%), and Transcutol^®^ (15–20%) (Table 1). The gel formulations were evaluated for pH, cohesiveness and adhesiveness, viscosity, drug diffusion, and pharmacokinetics. Viscosity was measured using a rheometer (DHR-3; TA Instruments, New Castle, DE, USA) equipped with a step-peltier stage (25 °C) and a 40 mm sand-blasted parallel steel plate. The following procedures were performed to determine viscosity. Samples (approximately 1 g) were placed on the plate and subjected to shear rates between 0.0001 and 100 s^−1^. Each sample was evaluated in triplicate. The data were fitted to the Herschel–Bulkley model to calculate the viscosity.

#### 3.2.5. Texture Analysis

A compression test was performed on the formulations using a Texture Analyzer TA.XT Plus (Stable Micro Systems, Surrey, UK). Measurements were performed at room temperature in triplicate. A cylindrical probe P/35 (35 mm diameter, aluminum) was used and an amount of 1 g of transdermal gel was delivered on the base. The probe compressed the formulation at a constant speed of 1 mm/s up to a 0.5 mm gap between the probe and the base and then returned to its start position.

#### 3.2.6. In Vitro Permeation Study

The in vitro permeation studies were carried out in a Franz Cell system (Hanson Research, Chatsworth, LA, USA) with a diffusion area of 1.767 cm^2^ and a volume capacity of 12 mL of the receptor medium. The receptor medium used was 12 mL phosphate buffer pH 7.4 containing 10% HPBCD to maintain the sink condition. The Franz diffusion cells were maintained at a constant temperature (32 ± 0.5 °C) through thermostatic bath circulation while the receptor medium was constantly stirred at 350 rpm by the magnetic bar during the experiments. Synthetic membrane/skin was carefully placed between the donor and receptor compartments followed by the addition of 1 mL DES saturated solution in phosphate buffer pH 7.4 (1.5 mg/mL) or 1 g gel into the donor chamber. Aliquots of 0.3 mL were collected at 0.5, 1, 2, 3, 4, 5, and 6 h and the medium was replaced. The samples were analyzed for permeated DES by HPLC. The cumulative amount of DES permeated through the membrane was calculated using the diffusion area.

#### 3.2.7. Skin Retention Study

After completion of the permeation study, the skin was removed from the Franz diffusion cells, washed thoroughly with water to remove the excess gel, and dried with a paper towel. The skin was cut into small pieces followed by the addition of 2 mL of methanol and 20 mM phosphate buffer pH 7.0 (80:20, *v*/*v*) as an extraction solvent. It was vortexed for 1 min and sonicated for 3 h at 25 °C. Samples were centrifuged at 3500 rpm for 5 min, filtered through a 0.45 µm syringe filter (Millipore^®^), and analyzed by the HPLC method. The retention of DES in pig ear skin was calculated by dividing the amount of the substances remaining in the samples by skin weight (μg/g).

#### 3.2.8. Pharmacokinetic Study

A comparative pharmacokinetic study was performed in New Zealand white rabbits (n = 4, male: female 1:1) weighing 2–4 Kg. The study was performed as per the Texas A&M’s Institutional Animal Care and Use Committees approved study #IACUC 2020-0048. The formulations used in the study were F8 transdermal gel and Clarinex tablets (5 mg DES). The animals were administered Clarinex 5 mg tablet by a pill popper (Emily pet pill disperser) followed by administration of 10 mL water. After a washout period of two weeks, the animal’s dorsal region was shaved and 100 mg gel formulation (F8 equivalent to 5 mg DES) was applied to a 3.1 cm^2^ area. The animals had free access to food and water and were monitored for any adverse events during the study. Blood (1 mL) was withdrawn through the central or marginal ear vein at 0, 0.5, 1, 2, 3, 4, 6, 8, 12, 24, 36, 48, and 72 h. A single dose of acepromazine 0.1–1 mg/Kg was administered intramuscularly to aid in vasodilatation prior to blood collection [50]. The blood samples were centrifuged at 13,300 rpm and 4 °C for 15 min to separate the plasma. The protein precipitation method was used to extract the drug from the sample. Methanol (900 μL) was added to plasma (300 μL) followed by the addition of 100 μL internal standard (IS) (DES-d4, 1 μg/mL) and 150 μL 0.1% formic acid solution, vortexed for 2 min, centrifuged at 13,300 rpm, and maintained at 4 °C for 15 min. The supernatant was analyzed for DES and IS by the UPLC–MS method.

#### 3.2.9. Ultra-Performance Liquid Chromatography Mass Spectrometry

The Acquity^®^ UPLC system (Waters Corporation, Milford, MA, USA) equipped with a quaternary pump, and PDA and QDa detectors were used in plasma sample analysis. The chromatographic separation of the samples was performed on Kinetex-C18 (3 × 100 mm, 2.6 μm) (Phenomenex, Torrance, CA, USA) in the stationary phase maintained at 35 °C. The mobile phase consisted of a mixture of 0.1% formic acid solution and methanol (60:40, *v*/*v*) flowing at 0.7 mL/min. Mass spectrometry parameters were electrospray positive ionization (ESI+) mode with 0.8 KV capillary voltage and 15 V collision energy. DES and DES-d4 masses were monitored by a QDa detector at 310.82 and 314.82, respectively. The linear range of DES was confirmed over the range of 2.5–100 ng/mL. The method was validated for linearity, selectivity, specificity, accuracy, precision, and the matrix effect.

## 4. Conclusions

Desloratadine has good permeation through the skin as suggested by almost immediate absorption and high flux values from the solution. Transdermal gel formulation exhibited a similar trend. Pharmacokinetic behavior of transdermal gel was completely different from the Clarinex tablet. Pharmacokinetic parameters of the transdermal gel (C_max_, AUC, t_1/2,_ λ_z_, and MRT) indicated that the gel formulation had higher bioavailability, and eliminated slowly compared to tablet formulation. The bioavailability of gel formulation was 2.4–3.2 fold of the tablet formulation. It is possible to further reduce the dose of the drug by the transdermal route. Thus, the long term metabolic effect of antihistamines may be reduced or eliminated by the transdermal route of administration of desloratadine.

## Figures and Tables

**Table 1:** Composition of desloratadine hydrogel formulations and their characterizations.

Formulation	Polymer (%)	Drug (%)	Transcutol^®^ (%)	Viscosity (Pas)	Flux- Ear Skin (μg/cm^2^.h)	Papp- Ear Skin (cm/h)	Flux- Strat-M^®^ (μg/cm^2^.h)	Papp- Strat-M^®^ (cm/h)
F1	2.0	2.50	20.0	31.9 ± 2.7	248.6 ± 31.4	1.24 × 10^−2^	12.6 ± 1.5	6.28 × 10^−4^
F2	3.0	2.50	20.0	243.9 ± 21.5	269.3 ± 35.8	1.35 × 10^−2^	34.7 ± 3.7	1.74 × 10^−3^
F3	2.5	3.75	17.5	180.9 ± 16.3	256.7 ± 27.4	8.56 × 10^−3^	36.4 ± 3.9	1.21 × 10^−3^
F4	3.0	5.00	20.0	374.5 ± 25.4	302.5 ± 38.2	7.56 × 10^−3^	38.2 ± 4.1	9.55 × 10^−3^
F5	3.0	2.50	15.0	493.4 ± 35.9	149.7 ± 17.9	7.49 × 10^−3^	24.5 ± 2.7	1.23 × 10^−3^
F6	3.0	5.00	15.0	519.9 ± 48.3	291.5 ± 30.5	7.29 × 10^−3^	36.5 ± 3.8	9.11 × 10^−3^
F7	2.0	5.00	15.0	104.6 ± 9.0	287.9 ± 31.6	7.20 × 10^−3^	46.9 ± 4.8	1.17 × 10^−3^
F8	2.0	5.00	20.0	65.5 ± 5.5	436.8 ± 44.2	1.09 × 10^−3^	97.4 ± 9.9	2.43 × 10^−3^
F9	2.0	2.50	15.0	58.2 ± 4.5	347.0 ± 36.9	8.68 × 10^−3^	51.7 ± 5.1	1.29 × 10^−3^

## Data Availability

Data is contained within the article.
